# The Prince of Wales's Hospital Fund for London

**Published:** 1899-03-04

**Authors:** 


### THE PRINCE OF WALES'S HOSPITAL FUND FOR LONDON.

### ANNUAL MEETING.

THE second annual meeting of the Prince of Wales's Hospital Fund for London was held on Thursday, 23rd ult., at Marlborough House, H.R.H. the PRINCE of WALES in the chair. There were also present: The Chief Rabbi, Viscount Duncannon, Lord Rothschild (treasurer), the Chairman of the London County Council (Mr. T. McKinnon Wood), the Chairman of the School Board for London (Lord Reay), the Governor of the Bank of England (Mr. Hugh Colin Smith), the President of the Royal Society (Lord Lister), the President of the Royal College of Physicians (Sir Samuel Wilks, Bart.), the President of the Royal College of Surgeons (Sir William MacCormac, Bart.), Sir Savile Crossley, Bart. (hon. secretary), Sir Trevor Lawrence, Bart., Sir Henry Burdett, K.C.B., Mr. A. G. Sandeman, Mr. Julius Wernher, the Master of the Clothworkers' Company (Mr. William Latham, Q.C.), and Mr. J. G. Craggs (hon. secretary).

The minutes of the meeting of the General Council held in December last having been read and confirmed,

Sir SAVILE CROSSLEY (hon. secretary) announced that letters regretting their inability to attend had been received from the following: The Bishop of London, the Bishop of Rochester, the Rev. T. Bowman Stephenson, Lord Rowton, Lord Iveagh, Lord Farquhar, Mr. John Aird, M.P., Mr. Sydney Buxton, M.P., and the Right Hon. C. Stuart Wortley, Q.C., M.P.

### THE REPORT OF THE COUNCIL.

Mr. HUGH COLIN SMITH (Chairman of the Executive Committee) read the report of the Council for the year 1898, which was as follows:—

"In submitting the following report of the operations of the Prince of Wales's Hospital Fund for London during the second year of its existence, it will be as well to recapitulate the policy adopted by His Royal Highness the President with the advice of the General Council. Briefly, it is to supplement the resources of those hospitals which, while doing good work, are most in need of assistance. The information on which the awards are based was obtained from the Visiting Committee appointed in the summer. This committee, of which Sir Trevor Lawrence was chairman, was composed in almost equal parts of surgeons and physicians of wide experience, and of business men specially acquainted with hospital management. The committee divided itself into sub-committees, to each of which was assigned a group of the hospitals situated within a radius of seven miles from Charing Cross. The report of the Visiting Committee provided the Distribution Committee with valuable and reliable information as to the needs, condition, and system of the various hospitals.

"The total sum distributed this year amounted to £32,500. Of this sum £28,200 has been given as annual grants, and the balance as donations. In the majority of cases the grants have been given on certain conditions. The effect of the action of the Fund will be that, in addition to supplying certain specified needs, the awards for 1898 will enable 57 closed beds to be reopened. The awards made in 1897 and 1898 have not only reopened, but will continue to maintain, 242 beds which were previously closed. This in itself is equivalent to building and maintaining another large hospital.

"Of the hospitals lying within a radius of seven miles from Charing Cross, almost all of those which applied for grants, in reply to advertisements in the London papers, have been assisted by the Fund.

"Not only has material financial help been afforded to the hospitals of London and the convalescent homes established for the benefit of Londoners, but these institutions have profited, and will continue to profit, by the inspection carried out and the recommendations made by the Visiting Committee. The governing bodies have had their attention called to defects in accommodation and management, and it may be confidently hoped that such representations may lead to the adoption of the highest standard on the part of all.

"The future work of the Fund must evidently depend on its financial condition. With regard to subscriptions there will occur, from time to time, losses from death and other causes. These losses, however, it is hoped will be made up by new subscriptions. It is difficult to estimate the donations with any approach to certainty. Judging, however, from the experience of the past twelve months, many of the donors of 1898 will repeat their donations in 1899. In addition to such renewed donations, it is hoped that new contributions will be received from areas of subscription hitherto untouched or imperfectly dealt with; and that the Fund will receive a share of the large amount of legacies bequeathed annually for charitable purposes.

"The policy of His Royal Highness, the President, with which the Council concur, is not to hoard money unduly by capitalisation. In order, however, to ensure the permanent utility of the Fund, the Council are anxious to increase the total invested capital to such an amount as will, with the addition of the annual subscriptions, secure a revenue sufficient to carry out in full the intentions indicated by the original letter of the Prince of Wales. With this view every class in the community is being carefully approached. In the opinion of the General Council too small a proportion of the middle and working classes seem to realise the opportunity which the Prince of Wales's Hospital Fund opens to them to contribute, according to their ability, to institutions from which they in their need are entitled to derive benefit. Many subscribe to the Sunday and Saturday Hospital Funds, with which his Royal Highness the President and the General Council are working in cordial harmony. Still there are hundreds of thousands of men and women able to give small sums who subscribe neither directly nor indirectly to the hospitals of London, through the Prince of Wales's, the Sunday or the Saturday Funds, or in any other way whatever. The claims of all these Funds are, by the action of the General Council and their supporters, gradually being brought home to the well-to do part of the population of London and the suburbs, who have hitherto, for one reason or another, not taken an interest in hospitals. It is hoped that the natural independence of the working classes will induce them to aid in the work by which they, and those dependent on them, profit when suffering from illness or the victims of accidents.

"The receipts of the Fund have been much smaller in 1898 than they were in 1897. It was not to be expected that it should be otherwise as the year 1897 was the Jubilee Year and was commemorated with special liberality on the part of the public by gifts of capital as well as contributions to revenue. It could not, therefore, be hoped that during the year 1898 these special gifts would be repeated. The Council trust, however, that the enthusiasm of 1897 will be replaced by steady and continuous support.

"Some disappointment on the part of those hospitals which were not included in the distribution made in December, 1898, was to be anticipated. To have divided the amount available for distribution among all the London hospitals would have resulted in an insignificant dole being allocated to each. The principle adopted by His Royal Highness on the recommendation of the General Council was simple. Invitation was given, by advertisements in the principal London papers to all hospitals which desired to benefit by the distribution to send in their application accompanied by certain information. Those hospitals which responded to the advertisement were visited by sub-committees of the Visiting Committee. After due and close personal investigation of their needs and merits, a report was submitted to the Distribution Committee, who in due course laid their recommendations before the General Council. Some hospitals were eliminated from the list of beneficiaries because their needs were comparatively small, others from various reasons which commended themselves to the Council. The final award was made after full consideration of all the facts which could be ascertained.

"In conclusion, His Royal Highness the President and the General Council desire to tender their sincere thanks to all those who have in various ways given their services to promote the work."

The Prince of Wales moved the adoption of the report.

Lord Rothschild seconded, and

The report was unanimously adopted.

### The Accounts.

Lord Rothschild (treasurer), in presenting the accounts for the year ended December 31st, 1898, said that he did not think there was anything in them which called for any special remark at his hands. The total receipts for the year were £39,270, whilst the expenditure was £34,960, of which the hospitals received £32,500. The expenses, therefore, of carrying on this great work were £2,460, or only 7 per cent. of the total expenditure. He thought great credit was due to Mr. Craggs and the other gentlemen who had given their services and worked so hard for the benefit of the hospitals. Next year he (the speaker) hoped to present His Royal Highness with a larger and more satisfactory balance-sheet in order that His Royal Highness might be enabled to give more money to the hospitals of London in which he took so keen an interest.

The Prince of Wales moved, and Lord Lister seconded, the adoption of the accounts, which was carried unanimously.

### Nomination of Council and Officers.

The Prince of Wales: I have now, on the termination of their year of office, to convey to all the General Council, the hon. treasurer, and the hon. secretaries my very best thanks for the services they have kindly given during the past year. I hope that all those who are in office now will continue to hold the same positions for the coming year. I am aware that Mr. Smith ceases his duties as Governor of the Bank of England in April, but I shall be much obliged to him if he will consent for another year to continue to hold the post of chairman of the Executive Committee, which he now occupies.

Mr. Hugh Colin Smith signified that he would have great pleasure in doing so.

The present members of the Executive Committee were also requested by the Prince of Wales to serve again for the current year.

### Votes of Thanks to the President.

Lord Rothschild, in proposing a vote of thanks to the Prince of Wales for presiding, said His Royal Highness had not only the thanks of the General Council of the Fund, but the thanks of the whole of the metropolis for the great good he had done for the hospitals and the citizens of London generally. He (Lord Rothschild) only hoped they would be able to get more subscriptions in coming years to bestow on the suffering and needy of the metropolis.

Mr. William Latham, Q C., seconded, and the vote was unanimously passed.

The Prince of Wales: I have to return you my best thanks, gentlemen, for your vote of thanks. My duties are very easy and light ones; and I cannot say how indebted I am to all of you for the support you have given me. I know you are as anxious as I am that this Fund should be a lasting one, and that as years go on the public may see that not only is it a scheme started to commemorate the Queen's Jubilee, but that it will be of lasting benefit to the people of the metropolis.

The meeting then terminated.

